# Developmental Processes and Mechanisms: Ages 0–10

**Published:** 2009

**Authors:** Robert A. Zucker, John E. Donovan, Ann S. Masten, Margaret E. Mattson, Howard B. Moss

**Keywords:** Children, youth, growth and development, psychological development, underage drinking, alcohol and other drug (AOD) use initiation, AOD use behavior, risk factors, protective factors, environmental factors, social–environmental factors, predictive factors, early AOD use onset, AOD expectancies

## Abstract

Little information is available on alcohol use in children up to age 10, although rates appear to be low. This age-group is not without risk, however. In fact, numerous nonspecific and specific risk factors for subsequent alcohol use are prevalent in childhood. Alcohol-nonspecific risk factors include externalizing and internalizing behaviors, as well as environmental and social factors (e.g., stress, physical abuse, or other aspects of social interaction). Nonspecific childhood factors (i.e., predictors) are being identified to target specific population subgroups for preventive interventions. These efforts have identified a variety of predictors of drinking onset during childhood or early adolescence that predict adolescent and young-adult problem drinking, as well as adult alcohol use and alcohol use disorders. Alcohol-specific risk factors also are being identified, including children’s beliefs and expectancies about alcohol, as well as childhood social contexts (e.g., modeling of alcohol use by parents, portrayal of alcohol use in the mass media, and growing up in a family with an alcoholic family member). Together, these specific and nonspecific influences play a heavy role in determining a child’s risk of or resilience to later alcohol use and related problems.

The development of alcohol use behaviors is an ongoing process that lasts through all stages of childhood, adolescence, and young adulthood. Each of these developmental changes is associated with particular developmental characteristics as well as with specific risk and protective factors that ultimately impact an individual’s alcohol use and its consequences. This article describes the developmental period from birth to the age of 10 and the relevance of that developmental period to the later use and problem use of alcohol. Beginning with an overview of normative human development for the age-group (i.e., development that is expected according to generally accepted norms), it then discusses and describes the risk and protective factors related to future use, as well as the factors of preadolescence that relate to precocious use.

## Normative Development for Ages 0–10: An Overview

The era from birth to age 10 includes all growth and development that occurs from conception through the prenatal experience, infancy, early and middle childhood to the beginnings of adolescence. It is perhaps the most remarkable of all developmental periods for the sheer extent of the changes that take place. During this timeframe, the structure, organization, and functions of the brain change dramatically. The fundamental systems for self-and social regulation and for adapting to the world develop. The characteristic ways in which individuals regulate their emotions, respond to stress, solve problems, communicate, perceive, learn, and manage their relationships with others develop. The child’s “personality” forms. Although change continues throughout life, by age 10, many of the child’s fundamental adaptive systems are in place and are relatively stable. (The developmental periods and transitions, key developmental contexts, and developmental tasks and issues for this age-group are summarized in the table.)

## Alcohol Use in the 0–10 Age-Group

Little information is available on alcohol use in the 0–10 age-group. Surveys of contemporaneous use are exceedingly rare, and retrospective recall by adolescents is not very reliable, with the age of first use generally increasing the older the adolescents are when questioned (Engels et al. 1997; Johnson et al. 1997). The little contemporaneous data that exist suggest that alcohol use under age 10 is very low but not zero. The Partnership Attitude Tracking Study (PATS), which is sponsored by the Partnership for a Drug-Free America, examines lifetime alcohol use. The most recent survey, composed of a national probability sample of almost 2,400 U.S. elementary school students, was conducted in 1999. It found that 9.8 percent of 4th graders (approximately age 10), 16.1 percent of 5th graders (approximately age 11), and 29.4 percent of 6th graders (approximately age 12) had taken more than a sip of alcohol in their lives (Donovan 2007). The annual PRIDE Surveys (www.PRIDESurveys.com) look at alcohol use in the past year rather than over a lifetime. The 2003–2004 summary of school district surveys performed across the United States (http://www.pridesurveys.com/customercenter/ue03ns.pdf) found that 4.2 percent of 4th graders, 5.6 percent of 5th graders, and 8.7 percent of 6th graders had consumed a beer in the past year. Slightly more had drunk wine coolers (4.4 percent, 6.7 percent, and 10.3 percent, respectively), and fewer reported drinking liquor in the past year (1.9 percent, 2.8 percent, and 5.2 percent, respectively). Although these data are based on a large sample of children from many school districts across the country, they were derived from a convenience sample rather than a representative national sample. Consequently, the level of bias contained in these estimates cannot be determined.

There is little evidence that children under age 10 experience alcohol abuse or alcohol dependence despite some clinical reports and anecdotal stories of childhood alcoholics (Famularo et al. 1985; Gordon 1913). Alcohol problems, as opposed to abuse or dependence, are somewhat more prevalent. Chen and colleagues (1999) reported, for example, that 4.8 percent of 5th graders in Baltimore, Maryland, already had experienced one or more problems with alcohol. Figures are not available for 3rd and 4th graders but can be expected to be lower.

The question of when youth initiate alcohol use is a critical one, because younger ages of onset are associated with an increased likelihood of developing both problem drinking in adolescence (Ellickson et al. 2003; Fergusson et al. 1994; Gruber et al. 1996; Hawkins et al. 1997) and alcohol abuse or dependence in adulthood (Grant and Dawson 1997; Hingson et al. 2006; Pitkanen et al. 2005). The differential risk between childhood alcohol use (age 12 and under) and early adolescent use (age 13 or 14) is not yet known, but studies have shown that early alcohol use is associated with problematic drinking in later years. Onset of alcohol use by ages 10–12 also is related to a variety of other problematic outcomes in grade 12, including absences from school, drinking and driving, and use of marijuana and other illegal drugs (Gruber et al. 1996).

The following are several basic themes that frame a useful perspective for understanding the timing, processes, and experiences of the preadolescent time period as they relate to the use and problem use of alcohol:

- Much of what causes alcohol use and abuse in youths is not specific to alcohol but is related to other problems in childhood, such as conduct problems, aggressiveness, impulsivity, antisocial personality disorder, and attention problems (Kendler et al. 2003; Tsuang et al. 1998; Zucker 2006).
- During the birth–to–age-10 timeframe, children learn about alcohol, including what it does and who uses it and why. They also develop expectations about its use. All these factors shape when children will use alcohol, under what circumstances, and to what extent.
- If a child under age 10 consumes alcohol, it may affect the child’s developing brain structures and functions, including those related to planning, appetite, reward, and emotional and behavioral control.
- Social–environmental factors play a role in alcohol use, such as the effects of family, peers, school, community, and culture. Social norms, for example, dictate when alcohol use is or is not acceptable.
- The multilevel, dynamic interplay of biological, psychological, and social processes shapes normal development as well as the risk for alcohol use and misuse (Masten 2007).

## Risk Factors in Childhood That Are Nonspecific to Alcohol Use

A number of nonspecific risk factors in the birth–to–age-10 group are related to the early onset of drinking and the development of alcohol problems and alcohol use disorders in adolescence or adulthood. These risk factors for subsequent alcohol involvement are nonspecific because they also influence a broad range of other behavioral problems. In fact, much of the causal structure underlying youthful alcohol use and abuse is not specific to alcohol (Kendler et al. 2003; Tsuang et al. 1998; Zucker 2006).

### Individual Factors That Influence Risk

Two significant developmental processes that emerge at birth are tied to the later development of alcohol-related problems. The first process involves emotion and its control; the second involves behavior and attention. The ability to display emotion at varying levels of intensity, and the capacity to regulate it based on level of arousal and context, are core temperamental characteristics of all human beings that are uniquely expressed in each child, with differences among children observable at birth. The second developmental process involves behavior and attention. Differences in activity level among infants exist prenatally and are obvious immediately after birth. Infants differ in how quickly they respond to touch, sound, and light as well as in how much they move. Likewise, they manifest differences in the degree to which they can maintain their attention on an object and shift that attention with the presentation of new stimuli.

Such differences in response signal differences in a child’s rudimentary behavioral regulation and control system and in a child’s attention regulation and control system. Learning, planning, and thinking ahead require the development of strong self-control systems. When the systems are weak, social and academic achievement become more difficult, and the risk for substance use disorders increases substantially. One theory on the development of alcohol use disorders proposes that the delayed or inadequate development of behavioral, emotional, and cognitive regulation is a central dysregulatory trait that plays an important role in the early emergence of substance use disorders (Tartar et al. 2004). This hypothesis is supported by a significant body of evidence (Caspi et al. 1996; Clark et al. 2005).

The developmental processes of emotional, behavioral, and attentional control also are related to alcohol use and its progression into problem use. As a result, researchers have attempted to identify traits that reflect the early development of these processes. Over the past two decades, data from several longitudinal studies (Caspi et al. 2002; Cloninger et al. 1988; Eron et al. 1987; Masse and Tremblay 1997; Mayzer et al. 2002) have provided convincing evidence of the existence of two traits arising very early in life that appear to be markers for early alcohol use, heavy use, problem use, and alcohol use disorder years later. The two traits that have been identified are externalizing behaviors and, to a lesser degree, internalizing behaviors. Furthermore, research has shown that these traits remain relatively stable throughout childhood and adolescence (Fuller et al. 2003; Olweus 1979) and that those individuals exhibiting the highest persistence of self-regulatory problems are the most likely to have the more chronic and severe forms of substance use disorder in adulthood (Biglan et al. 2004; Campbell et al. 2000).

Externalizing behaviors in children are behavior problems reflecting emotional, behavioral, and attention undercontrol directed outward toward others—that is, aggressiveness or refusal to comply with instructions, impulsivity, and attention deficits, which usually result in academic problems in school (Biglan et al. 2004; Campbell et al. 2000; Patterson et al. 1998). Children with externalizing behaviors often have the added problem of coming from a disadvantaged family. The parents of these children may have mental health or behavior problems, including alcohol abuse or antisocial personality. Poor discipline exists within such families, external resources are scarce (Buu et al. 2007), and the children often are described as exhibiting high negative emotionality or difficult temperaments. In late childhood and early adolescence, a percentage of these children disengage from school, start to associate with deviant peers, exhibit increasingly risky behaviors, and engage in delinquent behavior. Even before the transition to adolescence, these young people are at elevated risk for alcohol use and the behaviors associated with it, including early and risky sexual activities, truancy, and the like.

Internalizing behaviors constitute the second trait domain implicated by the longitudinal data on risk for underage drinking. These behaviors already are emerging in early childhood, but they are not as easily detected in this age-group because they largely are aspects of inner experience, like anxiety, sadness, and depression. Only shyness and social inhibition are more readily evident to the casual observer and, thus, have been more clearly linked to later problem-drinking outcomes (Caspi et al. 1996; Kellam et al. 1982). More generally, evidence is weaker for this internalizing pathway to early alcohol use than for the externalizing pathway, although there appears to be a link between adolescent depression and alcohol initiation (Kaplow et al. 2001). The evidence is considerably stronger, however, for an internalizing pathway to alcohol use disorder (Chilcoat and Anthony 1996). Other factors that can be identified in young children and have been associated with future alcohol problems include early childhood sleep problems (Wong et al. 2004) and deficits in attention (Molina and Pelham 2003).

### Environmental and Social Factors That Influence Risk

Environmental factors influence the development of risk as well as the development of protective factors. Stress, nurturance, physical abuse, observed family conflict, and other aspects of social interaction affect the brain by changing the development of neural networks or by producing hormones that alter that development. These early environmental experiences affect the attention regulation and control system. An increasing body of evidence suggests that they also play a role in the development of drinking behavior. Early stress, for example, affects the brain areas and neurochemical systems that are involved in impulse control and the brain’s reward circuitry. These systems can increase the risk for alcoholism by facilitating the onset of drinking, the maintenance of drinking behavior, and relapse.

An infant’s ability to display, as well as regulate, emotion reflects a process of social interaction between the infant and his or her caretakers (Zucker et al. 2000). The degree of attentiveness and responsiveness of the mother influences both the infant’s emotional display and emotional regulation. As the infant matures, the mother’s social environment, including her relationships with the father and with other adults in her support network, also have an influence (Eckenrode et al. 2001; Eiden et al. 2004), as does her own prior social experience, including a history of physical, sexual, or emotional abuse or other trauma. Fathers, too, make a contribution to this process, even early in the life of the child. Alcoholic fathers, for example, are less sensitive and express greater negative affect toward their children than do nonalcoholic fathers, which, in turn, lowers infant responsivity to the parents (Eiden et al. 2004). Paternal depression, antisocial behavior, and aggression also are associated with lower parental sensitivity.

Parental responsiveness to a child’s needs gradually increases the self-regulatory capacity of the child (Calkins 1994). Parents who are depressed, antisocial, or aggressive toward their children and who create a conflict-ridden family atmosphere reduce their children’s capacity to regulate and control their own behavior (Campbell et al. 2000). In addition, early exposure to alcohol and other drug use by parents and peers is a risk for underage alcohol use. By contrast, the most effective family environments for reducing externalizing behavior in children and adolescents (Campbell et al. 2000) and, ultimately, for reducing drug involvement in adolescence (Shedler and Block 1990), are those characterized by greater warmth, moderate discipline, and less stress.

### “Predictors” in Scientific Research

The term “predict,” as used in scientific research, does not have the same meaning as the word “predict,” commonly used outside of scientific circles to mean the capacity to foretell a specific event. Scientific prediction means that there is a greater likelihood of an outcome occurring in a group where the “predictor” is present than in a group where it is not. In scientific research, a predictor can predict more than one outcome. A predictor states that there is, for example, a relationship between those people who exhibit behavior A now and those people who will exhibit behavior B later. That prediction does not mean that every person who exhibits behavior A now will end up exhibiting behavior B later. It does mean that in the population exhibiting behavior A now, a greater-than-expected portion will exhibit behavior B later, but it is not possible to identify specific members of the population who will do so. A predictor, in other words, does not foretell an individual’s destiny. Therefore, a child with certain predictors for alcohol abuse will not necessarily abuse alcohol later in life, just as a child not predicted to abuse alcohol later may do so. The concept of prediction is important, however, because it makes it possible to target specific populations in which a substantial percentage is at risk for problem behavior and to intervene in those populations.

#### Predictors of Childhood-Onset Drinking (Initiation Before Age 13)

The low rates of alcohol use in children (Kaplow et al. 2002; Oxford et al. 2001; Sobeck et al. 2000) have precluded the conduct of extensive longitudinal studies focused on that issue. Instead, longitudinal research has investigated adolescent, young adult, or adult alcohol use. When childhood initiation has been studied, the focus has been on more general substance use (i.e., alcohol, tobacco, or marijuana use). Significant predictors of children’s substance use initiation include less parental monitoring, fewer parental rules, lessened parent–child attachment, single-parent families, parental tolerance of substance use, and parental drug abuse. Deviant peer affiliation, social skills deficits, peer drug use, and overactivity in the child also are predictors.

A study of children tested at ages 4–5, 9–10, and 14 (Baumrind 1985) found that less social assertiveness was associated with earlier ages of alcohol initiation for both sexes. For girls, earlier onset also was related to less parental responsiveness and to less encouragement of the child’s individuality at age 4 and to less parental monitoring and lower socioeconomic status at age 9. Earlier onset of alcohol use for boys was related to less parental encouragement of independence and individuality at age 4 and to less individuation and self-confidence at age 9. When a child used alcohol for the first time in the early years of elementary school, it was generally the result of an adult—usually a parent or close family member—introducing the child to the substance. Later ages of initiation generally resulted from peer introductions.

#### Childhood Predictors of Early Adolescent Drinking

A variety of studies involving both high-risk and general population samples have identified childhood predictors of early-onset alcohol use. (Because approximately half of the population has not used alcohol prior to age 15, use before that age is considered “early use” or “early-onset” drinking.) The Seattle Social Development Study (Hawkins et al. 1997) of high-risk youth found that at ages 10–11, the following factors predicted early-age initiation of alcohol use:

- White ethnicity;
- Greater parental drinking;
- Less bonding to school; and
- Having more friends who drink.

A study of high-risk boys from Pittsburgh (Clark et al. 1999) found that early-onset alcohol use through age 15 (using at least one standard drink per episode as the criterion) was predicted by antisocial disorder (i.e., conduct disorder and oppositional defiant disorder). Attention deficit hyperactivity disorder (ADHD) or negative-affect disorder (anxiety or mood disorder), however, were not predictors.

A community-based sample of high-risk families (Wong et al. 2004) identified the following predictors of alcohol use by ages 12–14:

- Parental alcoholism;
- Mothers’ ratings of early childhood sleep problems;
- Trouble sleeping; and
- Being overtired at ages 3–5.

A study of a lower-socioeconomic-status sample of boys from Montreal (Dobkin et al. 1995) found the following:

- Ratings of fighting and hyperactivity at age 6 and ratings of the boys’ aggressiveness and their friends’ aggressiveness at age 10 predicted the onset of drunkenness at age 13.
- Drunkenness by age 15 was predicted by teacher ratings of higher novelty seeking and lower harm avoidance at ages 6 and 10 (Masse and Tremblay 1997).

Studies of population samples suggest that the early initiation of alcohol use in these community groups is predicted by factors that are very similar to those found in studies of high-risk samples.

Based on 10- to 12-year-old abstainers selected from the Minnesota Twin Family Study, predictors of alcohol initiation at age 14 included conduct disorder, oppositional defiant disorder, and any externalizing disorder (McGue et al. 2001). Major depressive disorder and ADHD were not predictors. Another study using the same sample (King et al. 2004) found the same externalizing factors to predict regular use, at least one episode of drunkenness, and heavy drinking at age 14. Other studies confirm the Minnesota findings and add the following predictors:

- In the Ontario Child Health Study, children rated by teachers as having conduct disorder at ages 8–12 were more likely to be regular drinkers 4 years later (Boyle et al. 1993).
- In a birth cohort study of New Zealand children, conduct problems at age 8 predicted the usual intake of alcohol, maximum intake of alcohol, and alcohol-related problems experienced before the age of 15. These predictors held even after controlling for gender, family socioeconomic status, parental illicit drug use, and parental conflict, all of which also relate to later alcohol use (Lynskey and Fergusson 1995).
- In the same New Zealand study, childhood attention deficit behaviors were not related to alcohol behaviors and problems at age 15; these findings are similar to the results found by McGue and colleagues (2001).
- In the Finnish Twin Study, less parental monitoring and a poorer home environment assessed at ages 11–12 predicted alcohol use by age 14 (Rose et al. 2001).
- Also in the Finnish Twin Study, greater behavior problems and male gender predicted alcohol use by age 14 (Rose et al. 2001). Genetic analyses from this study indicate that shared environmental influences are more important than heredity in drinking initiation during early adolescence.
- In the Great Smoky Mountain Epidemiologic Study of Youth, predictors of initiating alcohol use within 4 years after initial testing at ages 9, 11, and 13 included greater depression, less separation anxiety, and greater generalized anxiety (Kaplow et al. 2001).
- Multiple studies have correlated early pubertal maturation in girls with early-onset alcohol use (Deardorff et al. 2005; Wiesner and Ittle 2002; Wilson et al. 1994). This correlation usually is explained by the girls’ affiliation with older, drinking peers, but other explanations are possible.

#### Childhood Predictors of Middle-Adolescent Drinking

Findings about childhood predictors of middle-adolescent drinking include the following:

- In the Woodlawn Study, teacher ratings of aggressiveness in 1st-grade African-American boys predicted more frequent use of alcohol at ages 16–17 (Kellam et al. 1982). That finding did not hold for girls. Similarly, a trend for shyness related to less alcohol use by boys but not by girls.
- Childhood symptoms of inattention measured at ages 5–12 predicted frequency of drunkenness and adolescent alcohol problems in a controlled study of children diagnosed with ADHD (Molina and Pelham 2003).
- Greater extraversion and deficits in reading achievement predicted earlier onset of regular drinking and drinking with negative consequences in a study of families at high risk for alcoholism because of a family history of alcoholism that included many relatives (Hill et al. 2000). These findings are in contrast to the findings from a large general population sample (Kellam et al. 1982).

#### Childhood Predictors of Adolescent Problem Drinking

Early childhood predictors of problem drinking in adolescence have been studied in longitudinal samples involving high-risk youth. Two such studies found the following:

- In the Seattle Social Development project, being male and initiating drinking at an earlier age were the strongest childhood predictors of problem drinking at age 16 (Hawkins et al. 1997). The effects of parental drinking, friends’ drinking, school bonding, perceived harm of drinking, and other age 10–11 predictors were mediated by age of initiation. In other words, the importance of each of these factors varied depending on the age at which the child started to drink.
- In the Michigan Longitudinal Study, a slower rate of increase in behavioral control from preschool through middle childhood predicted more drunkenness and more problem alcohol use in adolescence in a study of high-risk youth (Wong et al. 2006).

#### Childhood Predictors of Young Adult Problem Drinking/Alcohol Dependence

Longitudinal studies that have followed children into young adulthood to assess their experience with alcohol problems have identified a variety of childhood predictors based on individual differences and early contextual influences. These findings include the following:

- Aggressiveness at age 8 predicted problem drinking at age 26 for boys, but not for girls, in a sample of Finnish children (Pulkkinen and Pitkanen 1994).
- Poor response inhibition predicted early initiation of drunkenness and problem use even when conduct problems were controlled (Nigg et al. 2006).
- Childhood aggression at ages 5–10, expressed as anger, sibling aggression, noncompliance, temper, and nonconforming behavior, was related to DSM–III–R alcohol abuse at ages 16–21 in a New York community sample (Brook et al. 1992).
- A lower ability to concentrate and lower levels of school achievement at age 10 were related to hazardous use of alcohol prior to age 21 in a study of males from prenatal clinics in Sweden (Wennberg and Bohman 2002). “Hazardous use” consisted of public drunkenness and drunken driving ascertained from police register data and high levels of reported alcohol intake.
- Parental conflict over childrearing and maternal rejection of the child assessed when the child was age 3 were found in the New York Longitudinal Study to predict more severe alcohol involvement when the child was age 19 (Vicary and Lerner 1986).

#### Childhood Predictors of Adult Alcohol Use and Alcohol Use Disorders

Studies that link childhood data to follow-up data on alcohol use and alcohol use disorders (i.e, alcohol abuse and/or dependence) collected after young adulthood, although rare, found the following:

- Boys, but not girls, from a birth cohort study of children from Dunedin, New Zealand, who exhibited undercontrol by being impulsive, restless, and distractible at age 3, were twice as likely as control children to have a diagnosis of alcohol dependence at age 21 (Caspi et al. 1996).
- Low conscientiousness and high sociability ratings at age 12 related modestly to alcohol involvement at ages 40–50 in the Terman Life-Cycle Study (Tucker et al 1995).
- Higher teacher ratings of extraversion and lower ratings of emotional stability among Hawaiian elementary school children were associated with greater adult alcohol intake when followed up at the average age of 45 (Jahoda and Cramond 1972).
- Higher ratings on novelty seeking and lower ratings on harm avoidance and reward dependence at age 11 predicted more likely alcohol abuse at age 27 in a study of Swedish children (Hawkins et al. 1997). Evidence of alcohol abuse was based on registration with the Swedish Temperance Board, arrests for drunkenness, driving-while-intoxicated charges, or treatment for alcoholism.
- Measures of (poor) motor development in the first year of life related to the diagnosis of alcohol dependence at age 30 in the Danish Longitudinal Study of Alcoholism (Manzardo et al. 2005). Motor development was measured by muscle tone at age 5 days, an inability to sit without support at age 7 months, and the inability to walk at 1 year.
- Lower math achievement scores in 1st grade, lower ratings of shyness (boys only), and mother’s regular alcohol use (both genders) predicted a diagnosis of adult alcohol abuse or dependence at age 42 in a followup of African-American children initially studied in the 1st grade as part of the Woodlawn Study (Crum et al. 2006).
- Psychologist ratings of extroversion/aggression at age 4 correlated with frequency of intoxication at age 25, and ratings of extroversion/outgoing correlated with lifetime alcohol problems at age 36 in a Stockholm prenatal study (Wennberg and Bohman 2002).
- A conceptual model that integrates the following factors predicted DSM–IV alcohol abuse and dependence at age 21: internalizing disorders, externalizing disorders, male gender, delinquency, unclear family rules, poor family monitoring, less bonding to school, living in a neighborhood with more troublemakers, having antisocial friends, having friends who drink frequently, bonding to antisocial friends, higher intentions to use alcohol, and more favorable attitudes toward alcohol at age 10 (Guo et al. 2001).

## Alcohol-Specific Risk Factors in Childhood for Future Alcohol Involvement

A multilevel, dynamic interplay of biological, psychological, and social processes shapes normal development as well as risk for alcohol use. Research has identified a number of factors in the 0–10 age-group that specifically predict risk for later alcohol use and alcohol use disorders (as compared with nonspecific risk factors that predict a spectrum of problems, including alcohol use). A significant alcohol-specific risk factor is the age of initiation, especially of binge drinking. Childhood and early adolescent onset of alcohol use increases the likelihood of developing both problem drinking in adolescence (Ellickson et al. 2003; Fergusson et al. 1994; Gruber et al. 1996; Hawkins et al. 1997) and alcohol abuse or dependence in adulthood (Grant and Dawson 1997; Hingson et al. 2006; Pitkanen et al. 2005). It is not yet clear, however, whether childhood alcohol use (i.e., at age 12 and under) or early adolescent alcohol use (i.e., at age 13 or 14) is the greater risk factor.

Early-onset drinking is related to a variety of problematic outcomes in addition to later alcohol involvement. Onset of drinking by ages 10–12, for example, is correlated with absences from school, as well as marijuana and other illicit drug use in grade 12 (Gruber et al. 1996). Onset by ages 12–13 (7th grade) is related to more delinquent behavior, school problems, smoking, and illicit drug use in grade 12 and to smoking, illicit drug use, drug selling, and criminal behavior at age 23 (Ellickson et al. 2003). A follow-up study in grade 10 found that those who initiated drinking by the fall of 7th grade reported more recent drinking, drunkenness, and alcohol or drug problems and were more likely to have initiated sexual intercourse, to have had more than two sexual partners, and to have become pregnant or gotten someone pregnant compared with those who had not started drinking (Stueve and O’Donnell 2005).

### Development of Children’s Beliefs and Expectancies^1^ About Alcohol

As children develop, they become aware of alcohol as an object in their social environment through a variety of means. They observe alcohol use by parents and other adults in the immediate social environment, they see drinking paraphernalia (i.e., bottles, cans, six-packs, special glasses for wine and cocktails, etc.) and experience the smell of alcohol in its different forms. They also make observational contact via the media, seeing adolescent and adult actors on television, in movies, magazines, newspapers, advertising, and other forms of media. In the absence of the actual drinking experience, observational learning about alcohol is the major influence in determining children’s attitudes toward alcohol and the expectations they have about the effects of drinking. A number of studies show this influence is not inconsequential. As children grow older, they learn that alcohol produces changes in thinking, feeling, and behavior and that it has a role in social relationships. They discover who uses it and why and, ultimately, develop expectations about its use. To a significant degree, these cognitive variables influence when they will consume alcohol, how much they will drink, and when they will consider it appropriate to decline use.

By preschool, children have learned certain norms about alcohol use, namely, that adults drink alcoholic beverages, children do not, and men drink more than women (Zucker et al. 1995). By ages 6.5–7.5, children understand the concept of alcohol (Fossey 1994). Their attitude toward adults depicted drinking alcohol basically is neutral at age 6 but becomes more negative through age 10 (Fossey 1994; Jahoda and Cramond 1972). Between ages 10 and 14, these attitudes shift to become more positive (Aitken 1978). Children also learn about the behavioral effects of drinking and hold definite beliefs about it by age 10 (Fossey 1994; Gaines et al. 1988; Miller et al. 1990). These expectations about the effects of alcohol on drinkers generally are negative in childhood (Noll et al. 1990), but they become more positive as the children get older (Miller et al. 1990) and as they enter adolescence (Dunn and Goldman 1998).

### Childhood Social Contexts That Influence Drinking

In this age-group, parents are the major source of children’s exposure to alcohol use. Research over four decades consistently indicates that children are more likely to become drinkers at some point in their lives if their parents are drinkers. Self-reports of alcohol use among children correlate significantly with their perceptions of parental drinking (Jackson 1997). In fact, even preschool children are aware in some way of their parents’ level of drinking; this awareness translates into perceptions of the world as more or less peopled by adults who drink, such that 3- to 5-year-olds from heavier drinking homes are more likely to think that, when adults drink, they will be drinking alcoholic beverages (Zucker et. al., 1995). The fact that these preschoolers see men as drinking more than women, implying a personal knowledge of adult-cultural norms, suggests that even at very young ages, children have an acute understanding of the level of drinking around them.

Thus, parents increase the likelihood of their child’s drinking by modeling alcohol use, by making alcohol accessible in the home, by actively encouraging their child’s experimentation with alcohol, and also by covertly teaching them that alcohol use is an acceptable, possibly even desirable, behavior. When children are asked to name the source of their first drink, they overwhelmingly cite their parents or their home. For example, the 1993–1994 Bogalusa Heart Study found that 78 percent of the children in grades 3 through 6 who had ever tried alcohol took their first drink with someone in the family (Johnson et al. 1997). A recent community survey of children in Oregon (Andrews et al. 2003) found that few of the children who had ever tried alcohol had done so without their parents’ knowledge, especially in grades 1 through 4.

Children, like adolescents, also learn about alcohol use and its effects through their exposure to its portrayed use in movies, television, and advertisements. The alcohol industry spends over $1.6 billion a year on advertising, which it places in a variety of media, including radio, television, magazines, newspapers, and billboards (Bonnie and O’Connell 2004). Because magazine and radio audience data do not include children under age 12, it is not possible to measure childhood exposure to alcohol advertisements in these media. However, it appears likely that children under age 12 are less exposed to magazine ads because of their reading levels and their choice in reading materials. Generally, children favor books over magazines, or if they read magazines they prefer those with advertising restrictions. The same situation appears to exist for television advertising. A July 2005 Center on Alcohol Marketing and Youth report indicates that children ages 2–11 are exposed to less than half as many television alcohol advertisements as adolescents ages 12–20. As a result, they are underexposed relative to their prevalence in the overall population (Center on Alcohol Marketing and Youth 2005). Nonetheless, they still are exposed to some television advertising of alcohol products. Children ages 2–11 saw an average of 99.4 alcohol advertisements on television between January and October of 2004. Of those advertisements, 81 percent were for beer and ale, 11 percent for spirits, 5 percent for alcopops, and 3 percent for wine. Assuming a similar exposure rate across the years, the average child would have seen almost 1,200 television advertisements for alcohol products before the age of 12.

In addition to alcohol advertisements, children are exposed to portrayals of alcohol use in G- and PG-rated movies and on television shows in which characters use alcohol, often without apparent consequences. These programs are pervasive in primetime (8–11 p.m.) television when children may be watching. An estimated 71 percent of sampled primetime episodes from the 1998–1999 season portrayed alcohol use (Christenson et al. 2000). Approximately 38 percent of the TV-G–rated programs considered appropriate for most children depicted alcohol use. The majority of episodes characterized drinking as a positive experience rather than a negative one, with adverse consequences portrayed or mentioned in only 23 percent of the episodes.

Is childhood exposure to alcohol use in the media important? Significantly more research is necessary to determine the relationship between media exposure to drinking and children’s initiation of alcohol use. However, a greater awareness of beer advertisements was significantly related to greater intentions to drink as an adult in a study of 5th/6th-grade students (Grube and Wallach 1994). Awareness was defined as the ability to correctly identify brand names of still photographs from current television commercials. A study of 10- to 14-year-old nondrinkers found that their level of exposure to alcohol use in motion pictures predicted whether they were drinkers 1 to 2 years later (Sargent et al. 2006).

### Children in Alcoholic Families: A Special Early-Risk Population

Approximately 9.7 million children age 17 or younger were living in households with one or more adults who were classified as having a diagnosis of alcohol abuse or dependence within the past year, according to data from the National Longitudinal Alcohol Epidemiologic Survey (NLAES) (Grant 2000). Approximately 70 percent of these children were biological, foster, adopted, or stepchildren. Therefore, 6.8 million children, or about 15 percent of children aged 17 or younger, meet the formal definition of children of alcoholics (COAs). From the standpoint of socialization risk, these numbers reflect only acute exposure (i.e., within the past year) to at least one alcoholic adult. According to additional data from the NLAES survey, 43 percent of the under-18 population, or slightly less than half of all children, were exposed to either a current or previously alcoholic adult in their household at some time in their childhood. Approximately 30 percent of COAs under the age of 18 were similarly exposed, but even this represents an enormous population at high risk.^2^ COAs have an increased risk for earlier drinking onset (Donovan 2004) and earlier progression into drinking problems (Grant and Dawson 1997). They are between 4 and 10 times as likely as non-COAs to become alcoholics themselves (Russell 1990).

#### Genetic Risk Factors

Studies of children with an alcoholic biological parent who were raised by nonalcoholic adoptive parents (e.g., Sigvardsson et al. 1996) make it possible to separate genetic and environmental influences on the development of alcoholism. In such studies, the adopted children with a biological alcoholic parent were significantly more likely to develop alcoholism later in life than were control children without a genetic risk for alcoholism even though alcohol abuse had not been modeled in their home. Such studies indicate a genetic risk for alcoholism. Additional research will be required to isolate the specific aspects of genetic risk that result in alcoholism and to identify environmental factors that moderate that risk.

#### Factors Involved in Familial Transmission

The increased risk of alcohol involvement experienced by COAs is attributable to the following:

- Genetically transmitted differences in response to alcohol that make drinking more pleasurable and/or less aversive;
- The socialization effects of living in an alcoholic household; and
- Higher transmission of risky temperamental and behavioral traits that lead the COAs into greater contact with earlier and heavier drinking peers.
- Thus, familial alcoholism is a proxy for multiple risk factors, not all of which may be present for a given child.

High divorce rates in alcoholic families makes evaluating the level of socialization risk a complex task. Quantification of that risk requires not only knowledge of the length of exposure to the actively alcoholic parent but also the developmental period during which that exposure took place. Some developmental periods have the potential to produce greater vulnerability than others (Caspi et al. 1996). In addition, a substantial number of alcoholic men marry alcoholic women (Schuckit et al. 2002). In such cases, risk exposure is multiplied and includes, in addition to genetic risk, increased socialization risk associated with each parent and with the risk created by impaired marital interactions (Fuller et al. 2003). Indirect socialization effects also play a role because parental psychopathology is a risk factor for poorer parental monitoring (Chilcoat and Anthony 1996), which, in turn, increases the probability of the COA’s involvement with a deviant peer group, including earlier exposure to alcohol-and other drug-using peers.

The offspring of alcoholics have an increased risk for psychiatric comorbidities as well as for the development of an alcohol use disorder. Hence, COA status is a marker of increased risk for a variety of behavioral and cognitive problems. These include attention deficit disorder, behavioral undercontrol/conduct disorder, delinquency, lower IQ, poor school performance, low self-esteem, and others (West and Prinz 1987). Research strongly suggests that some of these non–alcohol-specific characteristics lead to both problem alcohol use and elevated risk for alcohol use disorders (Caspi et al. 2002; Donovan and Jessor 1985). For example, one community study of high-risk families (Wong et al. 2004) found that parental alcoholism was a significant predictor of early-onset alcohol use and drunkenness (both by age 14).

#### Fetal Alcohol Exposure

Prenatal exposure to alcohol is a potential risk factor for early-onset drinking and for later alcohol problems. Research shows that even relatively modest levels of alcohol intake can have negative effects on the developing fetus. The effects of alcohol exposure on fetal development are referred to as teratogenic effects. Unfortunately, it is not yet clear what, if any, level of alcohol intake is safe during pregnancy, but it is known that teratogenic risks can occur at levels of alcohol intake during pregnancy that are not symptomatic of maternal alcoholism. Depending on the level and timing of alcohol exposure and other factors, such as nutrition and genetic vulnerability, these effects can be growth related, neurological, structural, and behavioral, and can reflect a spectrum of alcohol-related neuro-developmental disorders called fetal alcohol spectrum disorders (FASD). The developmental effects of prenatal alcohol exposure have been extensively studied in both humans and animals (Brown et al. 1991; Donovan and Jessor 1985; Goldschmidt et al. 2004; Jacobson and Jacobson 2002; Mattson and Riley 1998; Olson et al. 1992; Rasmussen 2005; Streissguth et al. 1995). They include effects on response inhibition, attention, executive functioning, behavior, and school achievement, all of which are risk factors for later alcohol problems.

In a 1989–1990 study involving children from 464 families in which the mothers’ drinking during pregnancy reflected a spectrum of consumption levels, the researchers followed up with the children when they were age 14. The mothers’ alcohol intake during pregnancy significantly predicted negative consequences of alcohol use in adolescence defined as personal and social difficulties such as fighting and neglecting responsibilities. These findings held even when controlling for family history of alcoholism, current parental drinking, and several parenting variables. When prenatal alcohol exposure was controlled for statistically, a family history of alcoholism was not a significant predictor. A later followup of this sample found that prenatal exposure to alcohol and a family history of alcoholism predicted young adult (ages 21–24) scores on the Alcohol Dependence Scale (Baer et al. 2003).

## Developmental Unfolding of Risk and Resilience

Developmental pathways lead toward underage drinking and related problems of adolescence (risk), as well as away from them (resilience). Substantial evidence indicates that developmental patterns evident well before 10 years of age can be used to predict both later alcohol use and alcohol problems, suggesting that some children already have started down developmental paths leading toward early use and abuse of alcohol (Chilcoat and Anthony 1996; Donovan 2007; Zucker 2006). In some cases, high-risk pathways are so well-established that preventive interventions should be undertaken (Biglan et al. 2004). Even so, all “predictions” about the future alcohol involvement of children and adolescents more closely resemble probabilities. The actual pathway a given child or adolescent will take toward or away from problematic alcohol use is not certain. Many factors can intervene to change the pathway. In fact, there are children who appear to be on the same pathways who diverge in their decision to drink. Likewise, there are those on a developmental pathway away from problematic alcohol use who ultimately become involved in it. To better address underage drinking, it is important to understand the pathways that lead toward it as well as those that lead away from it.

### Risk Aggregation

Risk aggregation theory would suggest that if risk factors are present at the individual, familial, and neighborhood levels, they can accumulate to produce a risk level that moves the child toward behavioral problems including alcohol involvement. As the child grows older, the risk structure often includes peer networks that are high in aggression, negative mood, and substance use. As a result of this aggregation of risks the child is likely to develop the following:

- Positive expectancies about the use and abuse of alcohol and other drugs;
- Very early onset of alcohol use; and
- A stable repertoire of behaviors that is prototypical for the eventual emergence of abuse/dependence.

### Resilience and Risk: Key Developmental Pathways and Their Relevance to Underage Drinking

Two major risk pathways for underage drinking are the externalizing pathway (which is characterized by antisocial behavior resulting from inadequate emotional and behavioral self-control) and the internalizing pathway (which is characterized by emotional distress from anxiety, depression, and a shy/inhibited personality). These pathways were discussed in detail in the section on the emergence of behavioral and emotional dysregulation.

In contrast, the pathways of resilience, which result in positive outcomes despite adversity and reduce the risk of underage drinking, have been less well-defined. Children on a low-risk pathway with regard to early alcohol use have been characterized as successful in age-related developmental tasks throughout childhood. They also are more likely to have effective parents, good self-regulation skills, sound stress management, success and engagement at school, and they are more likely to associate with prosocial peers who engage in little risky or antisocial behavior. This low-risk pathway was documented in a community study of high-risk children (Zucker et al. 2003). The group termed “nonchallenged” initially had low levels of externalizing and internalizing traits and came from families characterized by lower genetic risk load and lower social adversity (i.e., less fighting, less parent divorce, and lower parental psychiatric difficulty). The pattern of adaptation for these nonchallenged children remains better than that of the other children from age 3 all the way to the early teens.

Another group of children also began with low levels of externalizing and internalizing traits, but they experienced higher adversity, alcoholic, and sometimes antisocial alcoholic home environments. These children were termed “resilient” (Zucker et al. 2003) because they showed a similar pattern of relative stability, with lower levels of impulsivity and aggressiveness from early childhood through early adolescence, even though they were being reared in high-adversity family environments. However, these children also showed some evidence of “weathering” with regard to internalizing traits. Anxiety, sadness, and depression were low during the preschool and early school years but rose to approach the levels of more vulnerable children by early adolescence. The authors suggest that long-term exposure to very high family adversity eventually eroded the positive outlook these children had when they were younger. At the same time, not all resilient children “weather out” of their initially positive adaptation. Under conditions where the initial adversity has been countered by positive social experience (e.g., the affection and nurturance of one parent despite the alcoholism of the other) individuals continue to show positive adaptation in early adulthood (Werner 1986).

## Protective Factors

Two types of positive factors have been identified in the literature on risk, competence, and resilience. Promotive factors are factors that generally are associated with better outcomes at various levels of risk or adversity across contexts. In statistical terms, they are main effects. Protective factors are those that are associated with better outcomes in the context of higher risk or adversity. In statistical terms, they are moderator effects (Sameroff 2000). Some factors, such as parenting, can be both promotive and protective. Substantial research documents that good-quality parenting acts as a promotive factor with regard to many positive developmental outcomes. By the same token, good-quality parenting also appears to play a special protective role in very risky or hazardous situations. Parenting, as with many of the most widely studied promotive and protective factors in human development, has various dimensions across a range from desirable to undesirable. Whereas good parenting can be a promotive and a protective factor, bad parenting can be a risk or vulnerability factor for underage drinking and many other problematic outcomes among children.

Promotive factors in the case of underage alcohol use are those that predict fewer problems. Protective factors, on the other hand, moderate the effects of risk or adversity. In the latter case, for example, a protective factor can result in lower-than-expected alcohol-related outcomes given the general level of risk for alcohol use or alcohol use disorders. For children living in poverty in bad neighborhoods, surrounded by deviant peers who encourage underage drinking, effective parenting may have protective effects beyond the generally positive effects of good parenting on child outcomes. Relatively few studies in the alcohol literature have focused on establishing moderators of risk, particularly in longitudinal analyses for children under the age of 10.

## Conclusion

This article has reviewed some of the major developmental processes and mechanisms operating in the 0–10 age-group as they relate to alcohol use and early problem use. Although most children under the age of 10 do not drink, they nonetheless are affected by a variety of forces that already are shaping their overall development and their future behavior with regard to alcohol use. Numerous risk, vulnerability, and protective processes already are at work. Some of these factors are not specific to alcohol use, whereas others are. The fascinating interplay of biological, psychological, and social processes that shape risk, as well as normal development, begins well before preadolescence, but becomes more obvious as puberty begins. The following article by Windle and colleagues examines the 10–15 age-group, when certain developmental processes become even more evident and alcohol use most often begins.

## Figures and Tables

**Table:** Developmental Periods and Transitions, Key Developmental Contexts, and Developmental Tasks and Issues of Children Ages 0–10

**Variable**	**Examples**
Developmental periods (key transitions)	Prenatal (conception) periods Infancy (birth) Toddler and preschool years (upright locomotion, preschool entry) Middle childhood (transition to elementary school)
Key developmental contexts	Prenatal environment Caregiver relationships Family, home, and neighborhood Daycare, preschool, and kindergarten Primary grades of elementary school Peer play and activity groups School classrooms and playgrounds Friendships Media (television, music, electronic toys and games, computers, and movies)
Developmental tasks and issues	Forming attachment bonds with caregivers Understanding and speaking the language of the family Understanding, speaking, reading, and writing the language of the culture/school Sitting, walking, skipping, and other developmental motor milestones Impulse control Compliance with rules of conduct Toilet training Playing with peers Acceptance among peers in key community or school contexts Adjustment to school Learning to read

SOURCE: Zucker et al. 2008.

## Footnotes

1 Expectancy is a term commonly used in the alcohol research literature to mean expectations.

2 The apparent discrepancy between 43 percent of all children and 30 percent of children of alcoholics being exposed to an alcoholic adult in their household is accounted for by a variety of factors, including parental divorce where the child lives with the nonalcoholic parent or child placement outside the home because of parental alcoholism.
